# Tspan protein family: focusing on the occurrence, progression, and treatment of cancer

**DOI:** 10.1038/s41420-024-01961-0

**Published:** 2024-04-22

**Authors:** Huhu Zhang, Qinghang Song, Kaiwen Shang, Ya Li, Liangqian Jiang, Lina Yang

**Affiliations:** 1 Department of Genetics and Cell Biology, Basic Medical College, Qingdao University, Qingdao, 266071 China; 2 Health Science Center, Qingdao University, Qingdao, 266071 China; 3 Department of Medical Genetics, Linyi People’s Hospital, Linyi, China

**Keywords:** Cancer metabolism, Cancer therapy, Oncogenes

## Abstract

The Tetraspanins (Tspan) protein family, also known as the tetraspanin family, contains 33 family members that interact with other protein molecules such as integrins, adhesion molecules, and T cell receptors by forming dimers or heterodimers. The Tspan protein family regulates cell proliferation, cell cycle, invasion, migration, apoptosis, autophagy, tissue differentiation, and immune response. More and more studies have shown that Tspan proteins are involved in tumorigenesis, epithelial-mesenchymal transition, thrombosis, tumor stem cell, and exosome signaling. Some drugs and microRNAs can inhibit Tspan proteins, thus providing new strategies for tumor therapy. An in-depth understanding of the functions and regulatory mechanisms of the Tspan protein family, which can promote or inhibit tumor development, will provide new strategies for targeted interventions in the future.

## Facts

- The Tspan protein family is strongly associated with the development and progression of cancer.
- Members of the Tspan family of proteins serve as biomarkers of cancer progression.
- The search for effective drugs that induce cancer death and inhibit the death of immune cells and normal tissue cells is a promising direction for future cancer therapy.

## Open Questions

- Which structural elements contribute to the dual role of Tspan protein family members in cancer development and progression?
- How does the Tspan protein family play a role in the biological processes of cancer? What are the primary mechanisms?
- Can drugs targeting Tspan proteins, either directly or indirectly, be effective in cancer treatment?

## Introduction

Tetraspanins (Tspans), first discovered in 1990, constitute a superfamily of 20–50 kDa transmembrane proteins that are widely expressed and distributed in multicellular organisms. They play crucial roles in both physiological and pathological processes, and exhibit highly conserved protein sequences within the family and across distantly related species [1]. Studies have demonstrated the widespread expression of Tspans across various tissues and cell types, with many cells expressing multiple Tspan members [2]. The Tspan protein family in humans consists of 33 members, ranging from Tspan1 to Tspan33, with specific tissue-specific expression patterns [3]. For instance, Tspan5 exhibits high expression in cortical regions and Purkinje cells [4], while Tspan7 is highly expressed in brain tissue and islets [5, 6], Moreover, Tspan26 represents a B-cell-surface antigen that demonstrates widespread expression in mature B cells [7]. Tspans can interact with their own or cell-surface molecules, including integrins, immunoglobulin superfamily proteins, proteases, growth factor receptors, and intracellular signaling molecules, forming complexes known as Tetraspanin-enriched microdomains (TEMs) [8]. TEMs regulate essential physiological processes, including cell signaling, adhesion, migration, proliferation, and differentiation [9].

Depending on the tumor type and the specific member of the Tspan protein family, Tspans can have either a promoting or a suppressing role in cancer. For example, Tspan1 and Tspan8 are upregulated in pancreatic cancer and colorectal cancer, respectively, and promote tumor cell invasion, migration, and autophagy [10, 11]. Tspan15 plays a pivotal role in promoting tumor cell proliferation by enhancing ERK phosphorylation, which in turn drives the expression and secretion of connective tissue growth factor (CTGF). This signaling cascade ultimately drives the proliferation of hepatocellular carcinoma (HCC) [12]. However, Tspan32 stands as a notable exception to the overall pro-tumorigenic role of Tspans. Its overexpression effectively impedes leukemia progression and impairs the proliferation of leukemia stem cells [13]. This highlights the complex and context-dependent regulation of Tspans in tumor development. Apart from these examples, other members of the Tspan protein family also influence tumor cell behavior and response to therapy, making them potential markers and targets of tumor development. It can offer novel strategies for targeted drug therapy. The majority of Tspan proteins play a promoting role in tumor occurrence and development. Consequently, the judicious utilization of Tspan inhibitors can yield improved strategies for clinical treatment and tumor prognosis.

## The molecular structure of the Tspan protein family

**Fig. 1:**
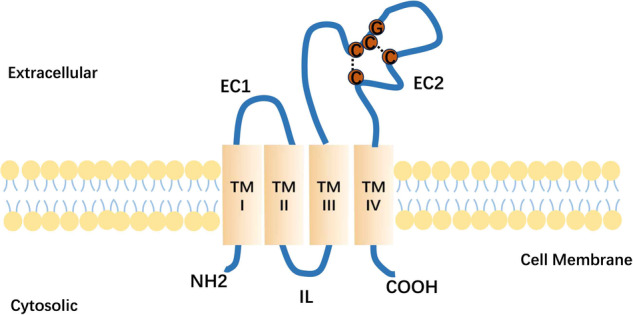
General structure of Tspan proteins. Tspan proteins include four transmembrane structural domains (TM), two short N-terminal and C-terminal cytoplasmic tails, and two extracellular portions called the EC1 and EC2 structural domains. EC2 is the functional region of the Tspan protein family and often contains a conserved CCG sequence.

Tspans consist of four highly hydrophobic and conserved transmembrane structural domains: TM1, TM2, TM3, and TM4. TM1 and TM2 are linked by a small extracellular loop (EC1) containing 20–28 amino acids, while TM3 and TM4 are connected by a larger extracellular loop (EC2) comprising 76–131 amino acids [14, 15]. TM3 are connected by a very short intracellular loop (IL), and Tspans include short cytoplasmic N-terminal and C-terminal ends [16]. Tspans have several distinct features from other proteins with four TMs. Firstly, the TM1, TM3, and TM4 structural domains feature polar residues of unknown function, which include an absolutely conserved CCG (Cys-Cys-Gly sequence) motif and two additional cysteine residues. These highly conserved amino acid residues constitute one of the most distinctive features that set Tspans apart from other proteins with four transmembrane domains [17]. In addition, more than 50% of the four-span proteins carry the PxxCC (Pro-x-x-Cys-Cys) motif, where x can be any amino acid [15, 18]. Cysteine pairs in EC2 play an important role in the correct folding of the structural domain by forming disulfide bridges (Fig. 1).

Each Tspans has specific partners, including integrins and histocompatibility antigens, etc. These form TEMs through protein-protein interactions, which enable the transmission of signals from extracellular, intermembrane, and intracellular proteins, directly or indirectly influencing intracellular signaling pathways to regulate cellular activities and metabolism [19–21]. The cytoplasmic structural domain contains palmitoylation sites on conserved intracellular cysteine residues. Specific functions are associated with the distinct structural domains of Tspans [22]. The transmembrane structural domains stabilize individual Tspans and facilitate binding to other Tspans on the cell membrane [22]. The variable region of EC2 mediates specific protein-protein interactions and the cytoplasmic region may provide a link to the cytoskeleton and signaling molecules [23]. Tspan5, Tspan10, Tspan14, Tspan15, Tspan17, and Tspan33 possess a unique property of containing 8 cysteines in their EC2. These proteins can undergo phosphorylation, methylation, and ubiquitination [18]. Palmitoylation of the EC2 of Tspan28 inhibits infection by the hepatitis C virus [24].

## The role of the Tspan protein family in cancer progression

**Fig. 2:**
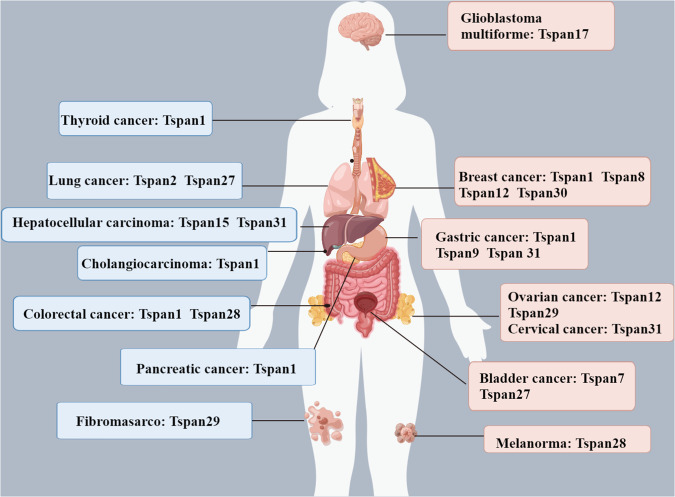
The distribution of Tspan protein family members in different cancers.

The progression of cancer involves a cascade reaction where cancer cells escape from the primary site, infiltrate blood vessels, circulate, and colonize distant sites. Accumulated evidence indicates that the Tspan protein family plays a regulatory role in various aspects of tumor biology, including cell proliferation, cell cycle progression, invasion, migration, autophagy, and apoptosis, mediated by signaling pathways [8, 14]. Furthermore, the members of the Tspan protein family exhibit distinct expression patterns across various types of cancers (Fig. 2). Despite the high structural conservation among members of the Tspan protein family and the absence of fundamental differences in the assembly of transmembrane and signal transduction molecules within TEMs, these proteins exert distinct regulatory roles in tumorigenesis. Specifically, they can either promote or inhibit the development of tumors (Table 1).

**Table 1:** The role of the Tspan protein family in cancer progression.

Member	Cancer type	Type cell	Expression patterns	Effect	Ref
Tspan1	Cholangiocarcinoma	HuCCT1, CCLP1, KMBC, RBE, HCCC-9810	mRNA and protein	Promotes the proliferation of tumor cells	[28]
	Breast cancer	MCF-7, T47D, MDA-MB-231, SK-BR-3	mRNA and protein	Promotes cell proliferation by activating PI3K/AKT signaling pathway	[29]
	Pancreatic cancer	SW1990, BxPC3, Capan1, PANC-1	mRNA and protein	After overexpression of Tspan1, migration and invasion ability were enhanced	[20]
	Colon cancer	SW620	Protein	Cell cycle arrest in G_1_/G_0_ phase	[40]
	Gastric cancer	SGC-7901, SUN-1, AGS, HGC-27, BGC-823	Protein	Tspan1 knockdown induces G_1_/G_0_ phase block	[41]
Tspan2	Lung cancer	A549	mRNA and protein	TSPAN2 enhances cell motility and aggressiveness	[47]
Tspan7	Bladder cancer	5637, T24, EJ	Protein	Inhibits cell proliferation by inhibiting p-PI3K and p-AKT	[33]
Tspan8	Breast cancer	MDA-MB-231, HCC-1954, MCF-7, T47D, BT474, MDA-MB-468	Protein	Promotes tumor invasion and migration	[48]
Tspan9	Gastric cancer	SGC-7901	mRNA and protein	Cells were stuck in G_1_ phase with a significant decrease in S-phase cells	[42]
Tspan12	Ovarian Cancer	SKOV3, A2780, OVCAR3	mRNA and protein	Tspan12 knockdown significantly slows cell cycle progression via G1-S-G2/M	[43]
	Breast cancer	MDA-MB-231	Protein	Promote tumor apoptosis	[57]
Tspan13	Thyroid cancer	TPC-1	mRNA and protein	The downregulation of Tspan13 significantly Promoted apoptosis	[21]
Tspan15	Hepatocellular carcinoma	HepG2	Protein	Promotes cell proliferation by activating EGFR/MAPK/ERK axis	[30]
Tspan17	Glioblastoma multiforme	U87MG, MT-330	mRNA and protein	Tspan17 knockdown promotes cell apoptosis	[58]
Tspan24	Epidermoid carcinoma	HSC5	mRNA and protein	Upregulation of Tspan24 accelerates tumor invasion and metastasis	[49]
Tspan27	Bladder cancer	YTS-1	Protein	Promote the proliferation of tumor cells	[31]
	Prostate cancer	PC-3	Protein	Promote the adhesion and migration of tumor cells	[50]
	Lung cancer	HSC-2, H1299	Protein	Inhibited the migration of tumor cells	[51]
Tspan28	Colorectal cancers	SW620, LOVO	mRNA and protein	Promote invasion and migration	[52]
	Melanoma	C8161, MelJuSo, SK-Mel-2, Malme-3M	mRNA and protein	Enhances the ability of tumor invasion and metastasis	[53]
Tspan29	Ovarian cancer	HTOA	mRNA and protein	Tspan29 reduces integrin expression	[35]
	Fibromasarco	HT1080	Protein	Overexpression of Tspan29 inhibited cell adhesion, migration, and proliferation.	[34]
Tspan30	Breast cancer	MCF-7	Protein	Inhibit cancer metastasis	[55]
Tspan31	Hepatocellular carcinoma	BEL-7402, SMMC-7721	mRNA and protein	Promotes the proliferation by activating the Akt/GSK-3β/β-catenin pathway	[32]
	Cervical cancer	HeLa, Siha	mRNA and protein	Suppress the cell cycle progression	[44]
	Gastric cancer	AGS, HGC-27, MGC-803, BGC-823, SGC-7901	mRNA and protein	Inhibits cell apoptosis	[59, 60]

### The role of the Tspan protein family in cell proliferation

The proliferation of cancer cells refers to the rapid division and multiplication of cancer cells under certain conditions, resulting in the formation of tumors [25, 26]. The proliferation of normal cells is regulated by the differentiation and death of organ cells as well as the regulation of signals, while the proliferation of cancer cells loses these self-regulatory abilities, which is considered to be the root cause of cancer [27]. According to the carcinogenic mechanism, the Tspan protein family is significantly associated with cancer proliferation in different cancers. Tspan further inhibits or promotes the ability of cancer cells to proliferate by inhibiting and activating signaling pathways related to cancer proliferation [28]. In other words, the Tspan protein family has two effects on cancer proliferation.

Tspan1 promotes the proliferation of cholangiocarcinoma and breast cancer by promoting the PI3K/AKT signaling pathway [28, 29]. Overexpression of Tspan15 in HepG2 significantly increased cell proliferation, which was achieved by activating the EGFR/MAPK/ERK axis [30]. In bladder cancer, the regulation of EGFR activity by Tspan27 is influenced by the ganglioside GD1a, which is important for the spatial organization of Tspan27-rich microstructural domains and interferes with Tspan27 recruitment of negatively regulated EGFR molecules such as tyrosine phosphatases, similar to the GD1a-Tspan27-EGFR complex, and the crosstalk between Tspan27, integrin α3 and HGFR intercrosstalk is also regulated by gangliosides. The ganglioside GM2-GM3-Tspan27 complex interferes with HGFR activation, accompanied by decreased GRB2 and HRA activity upstream of the MAPK pathway and GAB1 activity upstream of PI3K, thereby affecting cell motility and proliferation [31]. In HCC, Tspan31 promotes cell proliferation and motility by activating the AKT/GSK-3β/β-catenin pathway [32].

**Fig. 3:**
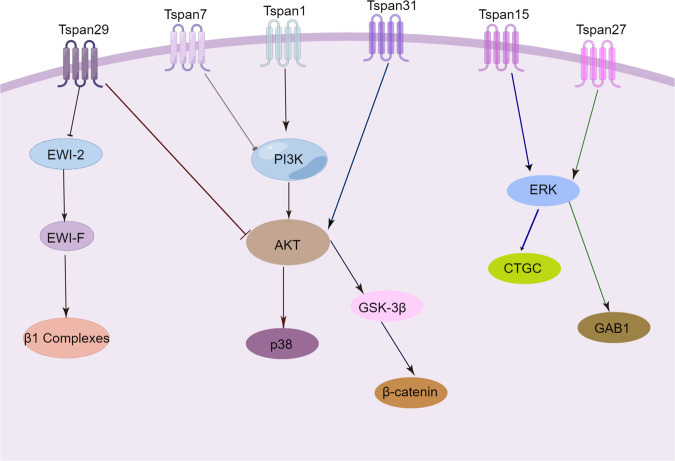
Signaling pathways of the Tspan protein family regulating tumor cell proliferation. The Tspan protein family has a dual role in cancer proliferation. Tspan1, Tspan15, Tspan27, and Tspan31 promote cancer proliferation, and conversely, Tspan7 and Tspan29 inhibit cancer proliferation.

Conversely, in bladder cancer, overexpression of Tspan7 inhibits p-PI3K and p-AKT, thereby inhibiting the proliferation of bladder cancer [33]. In ovarian cancer and fibromasarco, Tspan29 inhibits cell proliferation and migration by affecting several β1 integrin subpopulations or forming EWI-2/EWI-F/β1 complexes, inactivating AKT, p38, and EGFR signaling pathways [34, 35]. As the research on Tspan proteins deepens, there is more and more evidence that the Tspan protein family is involved in the regulation of tumor proliferation. Based on the dual role of the Tspan protein family for tumors, the study of Tspan on cancer proliferation, prevention, treatment, and prognosis can provide new ideas (Fig. 3).

#### Mechanism summary

Tspan1 and Tspan15 activate the PI3K/AKT and EGFR/MAPK/ERK pathways, respectively, leading to enhanced proliferation in cholangiocarcinoma and breast cancer. Tspan27 facilitates EGFR activity and cell motility through interactions with gangliosides and other proteins. Tspan31 promotes proliferation and motility in hepatocellular carcinoma via the AKT/GSK-3β/β-catenin pathway. In contrast, Tspan7 suppresses proliferation in bladder cancer by inhibiting p-PI3K and p-AKT. Tspan29 exerts anti-proliferative and anti-migratory effects in ovarian cancer and fibrosarcoma by targeting specific β1 integrin subpopulations and signaling pathways.

### The role of the Tspan protein family in the cell cycle

The intricate choreography of the cell cycle, encompassing Gap phases (G1, G2), DNA synthesis (S phase), and mitosis (M phase), is meticulously orchestrated by various factors, including the Tspan protein family. Their influence on this fundamental process is evident through their interactions with key regulatory components [36–38]. Malignant cells exhibit a distorted cell cycle, bypassing critical checkpoints that normally restrain excessive cell division. This deregulation enables cancer cells to evade normal growth constraints and acquire the ability to undergo limitless replication, forming tumors and metastasizing to distant sites [39]. The Tspan protein family plays an important role in the orderly operation of the cell cycle. In colon cancer, after Tspan1 is knocked down, the cell cycle is blocked in the G_1_/G_0_ phase [40]. Similarly, in gastric cancer, TSPAN1 knockdown induces G_1_/G_0_ phase block and inhibits invasion and migration [41]. In contrast, in gastric cancer, overexpression of Tspan9 significantly inhibited cell proliferation, with cells remaining in the G_1_ phase and a significant decrease in S-phase cells [42].

Mechanistically, Tspan proteins engage in a delicate tango with key orchestra conductors of the cell cycle. Tspan12 knockdown in ovarian cancer disrupts the rhythmic progression through G1-S-G2/M phases, implicating its involvement in the intricate interplay of cyclins and cyclin-dependent kinases (CDKs) like Cyclin A2, D1, E2, CDK2, and CDK4 [43]. This suggests that Tspan12 acts as a choreographer downstream of these critical cell cycle regulators. Likewise, in cervical cancer, Tspan31 directly silences CDK4 by targeting its 3′-untranslated region (3′-UTR), a master switch controlling cell cycle progression [44]. Furthermore, Silencing Tspan31 itself reverses this effect, highlighting its direct role in orchestrating the cell cycle tempo [44]. The downstream effects of the Tspan protein family usually serve to regulate the cell cycle. These findings paint a dynamic picture of Tspan proteins as context-dependent modulators of the cell cycle. While their downstream effects are evident, the precise mechanisms by which they interact with and manipulate specific signaling pathways remain shrouded in mystery. Future research endeavors that delve deeper into these intricate dances hold immense potential for unlocking the full therapeutic potential of Tspan proteins in the fight against cancer.

#### Mechanism summary

Knockdown of Tspan1 in colon and gastric cancer cells elicits G1/G0 phase arrest and inhibits cell proliferation, highlighting its tumor-suppressive potential. Tspan12 knockdown in ovarian cancer disrupts the rhythmic progression through G1-S-G2/M phases, suggesting its involvement in the intricate interplay of cyclins and CDKs, the orchestra conductors of the cell cycle. Tspan31 directly silences CDK4, a master switch controlling cell cycle progression, by targeting its 3′-untranslated region (3′-UTR) in cervical cancer, highlighting its ability to modulate cell cycle checkpoints.

### The role of the Tspan protein family in invasion and metastasis

Tumor cell metastasis is a multi-stage process in which malignant tumor cells spread from the primary site, through the body cavity, and vascular and lymphatic pathways, to reach other sites for further growth, and the process requires the participation of cell motility [45, 46]. Therefore, regulating the motility of tumor cells to prevent tumor cells from spreading and improve patient survival has also become one of the focuses of cancer treatment. Tspan1 regulates MMP2 expression through PLCγ and inhibits migration and invasion of pancreatic cancer cells [20]. In lung cancer, Tspan2 enhances cell motility and invasiveness by assisting Tspan8 to scavenge intracellular reactive oxygen species [47]. Tspan8 expression is upregulated in breast cancer stem cells, and upregulation of Tspan8 expression leads to increased drug resistance and stemness in tumor cells [48]. Mechanistically, Tspan8 enhances breast cancer cell stemness by interacting with PTCH1, recruiting ATXN3 deubiquitinating enzymes to inhibit proteasome-mediated degradation of the SHH/PTCH1 complex, and activating the Hedgehog signaling pathway. In human epidermoid carcinoma, Tspan24 binds to integrin α6 and EGFR and regulates laminin adhesion and migration [49]. By binding to EWI-2, Tspan27 inhibits laminin and fibronectin activity and suppresses prostate cancer invasion and migration [50]. Tspan27 interferes with lung cancer growth factor receptor (HGFR) signaling and thus activates Rac and CDC42, among a series of other mechanisms to inhibit cell invasion [51].

Tspan28 regulates the development of colorectal cancers through epithelial-mesenchymal transition, and inhibition of Tspan28 expression leads to reduced migration ability of cancer cells and reduced lung metastasis [52]. In addition, increased expression of Tspan28 in melanoma was also reported to promote tumor progression and metastasis [53, 54]. Tspan29 inhibits tumor metastasis, and its function is related to the inhibition of integrin-mediated motility. In ovarian cancer, Tspan29 expression levels correlated with integrins β1, α2, α3, α5, and α6, and downregulation of Tspan29 expression resulted in diminished stromal adhesion and diffuse growth [35]. Tspan30 inhibition of metastasis formation may be dependent on integrin endocytosis, MMP14 lysosomal degradation, and recruitment of tissue inhibitor of metalloproteinases 1 (TIMP1) [55]. Numerous studies have shown that most members of the Tspan protein family exhibit significant aggressiveness and metastatic properties. Therefore, inhibition of Tspan-related targets may provide new avenues to mitigate cancer metastasis and improve patient survival.

#### Mechanism summary

Tspan proteins interact with a wide range of signaling pathways, including integrin signaling, ECM remodeling, and angiogenesis, to orchestrate metastasis. Tspan1 regulates MMP2 expression and either promoting or inhibiting invasion. Tspan2 assists Tspan8 in scavenging intracellular reactive oxygen species. Tspan8 upregulation promotes breast cancer stemness and drug resistance.Tspan24 binds to integrin α6 and EGFR. Tspan27 binds to EWI-2, inhibiting laminin and fibronectin activity. It also interferes with HGFR signaling, activating Rac and CDC42.Tspan28 regulates through EMT. Tspan29 inhibits integrin-mediated motility. Tspan30 depends on integrin endocytosis, MMP14 lysosomal degradation, and recruitment of TIMP1.

### The role of the Tspan protein family in cell apoptosis

Apoptosis is the autonomous, orderly death of cells that are genetically controlled, also known as programmed death [56]. The Tspan protein family has a dual function in cancer cell apoptosis, namely anti-apoptotic and pro-apoptotic activities [21, 57]. Depending on the tumor type and the specific member of the Tspan protein family, the effect of Tspan proteins on apoptosis can be either promoting or suppressing cancer. For example, in breast cancer, Tspan12 has a pro-apoptotic function, as it significantly reduced primary tumor xenograft growth, while increasing tumor apoptosis [57]. Tspan12 removal also altered the expression of several genes regulated by β-catenin (CCNA1, CCNE2, WISP1, ID4, SFN, ME1), inhibiting alterations in tumor growth and metastasis [57]. Similarly, in thyroid cancer, the downregulation of Tspan13 significantly inhibited the proliferation and promoted the apoptosis of TPC-1 cells [21]. On the other hand, in glioblastoma multiforme (GBM), Tspan17 has an anti-apoptotic function, as it is associated with poor prognosis in GBM patients. Tspan17 knockdown promotes cell apoptosis [58]. Likewise, in gastric cancer, Tspan31 has an anti-apoptotic function, as the level of apoptosis in gastric cancer cells was significantly increased after upregulation of Tspan31 expression and significantly decreased after downregulation of Tspan31 expression [59]. Tspan31 facilitated the proliferation and metastasis of gastric cancer through METTL1/CCT2 and PI3K/AKT [59, 60]. Elucidating the molecular mechanisms of the Tspan protein family in apoptosis can help identify new therapeutic targets and develop new treatment strategies.

#### Mechanism summary

Tspan proteins interact with various apoptosis signaling pathways, including β-catenin, METTL1/CCT2, and PI3K/AKT, to orchestrate apoptosis. Tspan12 removal reduces tumor growth and promotes apoptosis, altering the expression of β-catenin-regulated genes. Tspan13 inhibits proliferation and promotes apoptosis. Tspan17 promotes apoptosis, potentially improving prognosis. Tspan31 inhibits apoptosis and increases proliferation and metastasis through METTL1/CCT2 and PI3K/AKT signaling pathways.

### The role of the Tspan protein family in cell autophagy

Autophagy is a process by which a cell engulfs its own cytoplasmic proteins or organelles and encapsulates them into vesicles and fuses with lysosomes to form autophagic lysosomes that degrade their encapsulated contents, thereby achieving the cell’s own metabolic needs and the renewal of certain organelles [61, 62]. Only Tspan1 and Tspan9 of the Tspan protein family have been reported to be associated with autophagy in cancer. Tspan1 expression was demonstrated to be upregulated in pancreatic cancer in vitro and in vivo, and Tspan1 deletion decreased the proliferation of pancreatic cancer cells. In addition, Tspan1 is a novel positive regulator of autophagy characterized by reduced expression of LC3-II and SQSTM1/p62 and inhibition of GFP-LC3 autophagic vacuoles [10]. In gastric cancer, Tspan9 knockdown was followed by a significant decrease in the LC3-II/I ratio and a significant increase in SQSTM1/p62 levels [63]. Tspan9 overexpression increased the LC3-II/I ratio and decreased SQSTM1/p62 levels [63]. The Tspan protein family has been less studied in cancer in relation to autophagy. Although it has been shown that Tspan proteins play an important role in cellular autophagy, an in-depth understanding of the effects of other Tspan protein family members in different cancers would make an essential contribution to the field of cancer therapy.

#### Mechanism summary

Tspan1 acts as a positive regulator of autophagy, characterized by reduced expression of LC3-II and SQSTM1/p62 and inhibition of GFP-LC3 autophagic vacuoles. Tspan9 knockdown decreases the LC3-II/I ratio and increases SQSTM1/p62 levels, while overexpression of Tspan9 increases the LC3-II/I ratio and decreases SQSTM1/p62 levels.

## The role of the Tspan protein family in tumor immunity

**Fig. 4:**
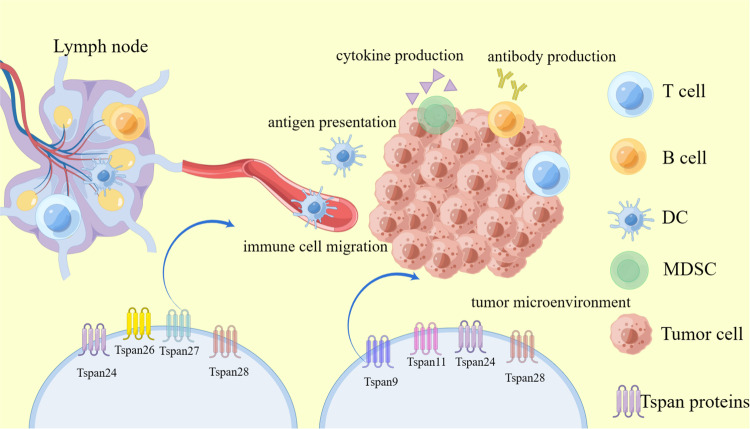
The role of Tspan protein family in tumor immunity. During tumor immunity, immune cells migrate from peripheral lymphoid organs to the tumor with the aim of destroying tumor cells, and the Tspan protein family is involved in this process; however, tumors can also undergo immune escape to evade immune surveillance, and tumor cells recruit different types of cells (e.g., Tregs and MDSCs) to create specific tumor microenvironments, and tumor microenvironments are composed of several different cells, including tumor cells and different immune cells, immune cells produce different soluble factors, including cytokines and antibodies, Tspan can produce immune-cell-mediated antitumor or pro-tumor immune effects in the tumor microenvironment.

Antitumor immunity is formed by different types of immune cells present in the tumor microenvironment, and there is growing evidence demonstrating the involvement of the Tspan protein family in tumor-associated immune responses [64]. Tspans can promote or inhibit tumor invasion and metastasis by interacting with the tumor cell microenvironment, Tspans induce immune responses to tumors in a context-dependent manner, and they can directly influence immune-cell signaling by interacting with key immune receptors (e.g., MHC molecules, CD4, CD8, CD19), Tspan protein family directly impacts immune-cell signaling, influencing antigen presentation, immune-cell migration, cytokine production, as well as T cell proliferation, activation, and subpopulation differentiation (Fig. 4).

The initial stage of the immune response involves the uptake, processing, and presentation of tumor antigens by APCs. Tspan25, Tspan26, Tspan27, and Tspan28 have demonstrated associations with the MHC class II complex [65]. Conversely, Tspan30 has been reported to inhibit antigen presentation [66]. Adequate immune responses require the migration of immune cells from the surrounding tissues to the tumor site. TSPAN proteins have been observed to influence dendritic cell migration [67]. Specifically, Tspan26 and Tspan28 have been shown to promote dendritic cell migration [68, 69], whereas Tspan27 inhibits dendritic cell migration68. Additionally, Tspan24 facilitates T cell migration [70]. The Tspan protein family can produce immune-cell-mediated antitumor or pro-tumor immune effects. Tspan28^−/−^ mice are severely impaired in tumor growth and metastasis, and lacking Tspan28 are defective in the inhibitory capacity of both Tregs and MDSCs [54]. Tspan7 is highly upregulated in tertiary lymphoid structures-related vessels and Tspan7 plays a role in lymphocyte stasis and lymphocyte recruitment involved in tertiary lymphoid structures-related vessels [71]. Tspans were also found to reduce infiltration of tumor immune cells, enrich immune-related signaling pathways, and express immune checkpoint proteins in cancer patients [72]. Tspan9 expression was significantly negatively correlated with tumor immune-cell infiltration and the immune checkpoint CTLA4, Tspan9 can decrease tumor immune-cell infiltration, enrich immune-related signaling pathways, and express immune checkpoint proteins to exert its tumor suppressor effects [72]. Tspan11 is a tumor mesenchymal-associated biomarker that correlates with tumor immunity [73]. Tspan24 can regulate the interactions between tumor cells and immune cells, and Tspan24 has the function of regulating both innate immune cells and acquired immune cells [74]. Tspan24 peptide tumor vaccine prepared with this inspiration also has potential therapeutic value. Tspan24 peptide tumor vaccines also have potential therapeutic value, and antitumor active immunity initiated by Tspan24 peptide may be an effective and safe method to inhibit tumor progression. Using the synthetic Tspan24 peptide as a tumor vaccine, the vaccine triggered an active immune response in both primary and metastatic tumor models, promoted tumor CD8 lymphocyte invasion and reduced MDSCs, and effectively inhibited tumor growth and metastasis [75]. Tspans affect immune function through lateral interactions with their counterparts, GPR56 (Gprotein-coupled receptor56) was found to be an inhibitory receptor expressed on human NK cells, and cis interaction with Tspan28 attenuates the cytotoxic activity of NK cells [76].

The Tspan protein family has the ability to impact cytokine production. Tspan27 assumes a crucial role in shaping innate immune signaling, specifically in the transport and signaling of Toll-like receptor 9 (TLR9). TLR9 is responsible for recognizing unmethylated cytosine-phosphate-guanine (CpG) motifs found in viral, bacterial, and fungal DNA. Notably, Tspan27’s regulation of TLR9-dependent nuclear translocation of NF-κB is essential for the production of inflammatory cytokines [77]. The Tspan protein family is associated with T cell proliferation; activation and subpopulation differentiation, and the Tspan1 protein is strongly associated with the polarization ratio of CD4 T cells and Th17 cells [78]. Tspan25 is a chaperone of the T cell-surface tyrosine phosphatase CD45 and can regulate the CD45 activity, Tspan25 is required for T cell activation and proliferation [79]. In conclusion, different Tspans can play positive or negative regulatory roles in innate and acquired immunity during tumor immune surveillance. Further exploration of the Tspan protein family’s role in immune cells will deepen our understanding of these membrane proteins’ contributions to antitumor immunity and their potential as targets for immune-cell-based interventions [80].

### Mechanism summary

Tspan25, Tspan26, Tspan27, and Tspan28 interact with the MHC class II complex, influencing antigen presentation by antigen-presenting cells (APCs). Tspan26 and Tspan28 promote dendritic cell migration, while Tspan27 inhibits dendritic cell migration. Tspan24 facilitates T cell migration. Tspan27 plays a crucial role in innate immune signaling, specifically in the transport and signaling of Toll-like receptor 9 (TLR9), which is responsible for recognizing unmethylated cytosine-phosphate-guanine (CpG) motifs found in viral, bacterial, and fungal DNA. Tspan1 is strongly associated with the polarization ratio of CD4 T cells and Th17 cells. Tspan25 is a chaperone of the T cell-surface tyrosine phosphatase CD45 and regulates CD45 activity, which is required for T cell activation and proliferation.

## The Tspan protein family in exosomes

The Tspan protein family is prominently present in the membranes of exosomes and extracellular vesicles [81]. Exosomes are small extracellular vesicles that cells secrete to facilitate intercellular communication by transferring cellular cargoes, including functional proteins, metabolites, and nucleic acids, to recipient cells [82]. Proteomics studies have revealed the widespread presence of the Tspan protein family in exosome membranes, where they can serve as targeting molecules for cell membrane binding or exert indirect control over cellular interactions through exosomes. Different tspan-associated proteins may be involved in the attachment process to specific proteins on target cells [83] These proteins play significant roles in physiological processes, including signal transduction, cell activation, motility, adhesion, and tissue differentiation [84]. The different roles that Tspan proteins can play in tumor progression may be the result of heterogeneous expression of Tspan proteins in exosomes [85]. An increase in Tspan29 and Prostate Specific Membrane Antigen (PSMA) double-positive plasma-derived circulating vesicles has been reported in patients with advanced metastatic prostate cancer, whereas double-positive Tspan29 and Tspan30 extracellular vesicles (EVs) are significantly increased in patients with limited prostate cancer [86]. Tspan proteins sorting and delivery to exosomes is tightly regulated. During advanced endosome stages, Tspan30 becomes enriched within luminal vesicles and is subsequently secreted as exosomes through the fusion of endosomes with the plasma membrane [55], The Alix and ESCRT-III pathways actively facilitate the sorting and delivery of Tspan proteins into exosomes. Additionally, Alix promotes the secretion of exosomes containing Tspan28, Tspan29, and Tspan30 [87].

The Tspan protein family exhibits diverse functions within exosomes. On one hand, it participates in morphogenesis, division, and fusion processes [88]. Tspan8 is able to modulate the content and function of EVs in breast cancer, and Tspan8 mediates a several-fold increase in the number of EVs in cell culture and the circulation of tumor-bearing animals [89]. Using breast cancer cells to obtain Tspan8-enriched small extracellular vesicles (SEVs), Tspan8 facilitated the binding of breast cancer cell-derived SEVs to target cells by enhancing the restriction of spreading and inducing restriction of fibroblasts. The binding or uptake of Tspan8-SEV promotes cellular motility and invasion and may be related to the FAK-SRC signaling pathway, EMT induction, and the upregulation of proteases in target cells [83]. On the other hand, multiple subpopulations of extracellular vesicles carrying distinct Tspan proteins are present in human blood, with varying levels of Tspan28, Tspan29, and Tspan30 across these subpopulations [90]. This variability enables Tspan proteins to serve as markers for exosome isolation and quantitative analysis [91]. Plasma EVs Tspan9 can be used as a marker for the detection of colorectal cancer [92]. Additionally, the heterogeneous expression of Tspan in exosomes allows them to determine the tumor type of a cancer patient with an unknown source of the primary tumor, supporting a diagnosis that leads to a more specific treatment plan for the cancer patient [93]. In addition, Tspan6 was found to be a negative regulator of exosome release, supporting lysosomal degradation of Syndecan4 and Syntenin, inhibiting the shedding of the extracellular structural domain of Syndecan4, and also having a positive effect on exosome formation or cargo composition [94]. The current study of exosome biology shows that Tspan6 is a negative regulator of exosome release, supporting lysosomal degradation of Syndecan4 and Syntenin, and inhibiting the shedding of the extracellular structural domain of Syndecan4. Exosomes may also provide some ideas for tumor therapy; hypoxic tumor cells release exosomes that induce tumorigenesis by promoting metastasis, angiogenesis, and modulation of the immune response, and targeting the biogenesis of these exosomes may be a therapeutic opportunity to reduce tumorigenesis, in addition, exosomes can act as drug delivery systems to transfer therapeutic compounds to cancer cells [95].

The main mechanisms of exosome biology have now been elucidated, but much remains unclear about the regulatory processes, and the heterogeneity of exosomes, their differential content, and their properties affected by donor and recipient cells add to the complexity of unraveling the regulatory processes [82] Despite the rapid development in the field of exosomes, the detailed understanding of the regulation and function of exosomes is still insufficient, the exact role of Tspan in exosome biogenesis is unclear, and there are still many challenges to be faced in the clinical application of tumor diagnosis and therapy.

### Mechanism summary

The Tspan family plays a multifaceted role in regulating exosome biogenesis, function, and targeting, thereby shaping their impact on cellular processes. Tspan30, undergoes a complex sorting process, guided by the ALIX and ESCRT-III pathways, ensuring their efficient incorporation into exosomes.Tspan8, contributes to the unique composition of exosomes, influencing the cargo they carry and their functional properties.Tspan6, modulates exosome formation and dynamics, ensuring their timely release and regulating their interaction with recipient cells.Tspan8-enriched exosomes exert pro-invasive effects on target cells, potentially through the FAK-SRC signaling pathway, promoting their migration and invasion. Tspan29, Tspan30, and Tspan9, in exosomes, provide potential biomarkers for distinguishing tumor types, aiding in diagnosis and prognosis. Exosomes released from hypoxic tumor cells promote tumorigenesis, and targeting the biogenesis of these exosomes can reduce tumor progression. It can also be employed as drug delivery vehicles to cancer cells.

## The role of the Tspan protein family as a biomarker in cancer diagnosis

The Tspan protein family exhibits variable expression levels across different cancer types, making it a potential biomarker for cancer (Table 2). Tspan1, Tspan11, Tspan13, Tspan28, Tspan29, and Tspan30 have been identified as biomarkers for tumor diagnosis and prognosis, while Tspan8, Tspan24, and Tspan27 show potential as biomarkers [96–99].

**Table 2:** Tspan protein family as a biomarker in cancer diagnosis.

Member	Cancer type	TSPAN protein expression	Reference
Tspan1	Pancreatic cancer	High expression	[96]
Tspan8	Breast cancer	High expression	[103]
	Ductal adenocarcinoma of pancreas	High expression	[103]
	Colorectal cancer	High expression	[11]
	Ovarian cancer	High expression	[97]
Tspan11	Breast cancer	Low expression	[73]
Tspan13	Breast cancer	Low expression	[98]
Tspan24	Prostate cancer	High expression	[99]
	Ovarian cancer	High expression	[97]
Tspan27	Breast cancer	Low expression	[105]
Tspan30	Breast cancer	Low expression	[108]

Tspan1 and CA2 serve as key genes for predicting prognosis and immunotherapy efficacy [100]. Tspan1, identified as a HUB gene closely associated with pancreatic cancer [101], exhibits significant correlations with tumor histological grade, T stage, clinical stage, and overall survival [102]. Tspan8 can promote the growth and migration of colorectal cancer cells [11]. Compared with normal adjacent tissues to cancer (NATs), the mRNA level of Tspan8 in pancreatic ductal adenocarcinoma, colon cancer, hepatocellular carcinoma, prostate cancer, rectal cancer, and gastric cancer is significantly increased [103]. Tspan24 serves as a favorable prognostic indicator in invasive lobular breast cancer and endometrial carcinoma. Conversely, its overexpression in gastric cancer, prostate cancer, and non-small-cell lung cancer indicates a poor prognosis. Circ_0020710, a CircRNA derived from Tspan24, promotes the proliferation, migration, and dissemination of melanoma cells, induces melanoma immune escape, and exhibits significant upregulation in melanoma. It is involved in various regulatory mechanisms, including ceRNAs, protein interactions, and the regulation of gene transcription and translation [104]. Tspan27 functions as a metastasis suppressor gene that is frequently downregulated in advanced stages of cancer [105]. Overexpression of Tspan27 can inhibit the invasion and migration of lung cancer cells. Tspan29 is sometimes employed as a biomarker for invasion and advanced stages of cancer, particularly in metastatic renal clear cell carcinoma, where it not only distinguishes tumor subtypes but also predicts the metastatic potential of renal cell carcinoma. In mesothelioma, the expression of Tspan29 is associated with survival and may serve as a favorable prognostic marker for patients [85]. Comparative proteomics analysis using a high-performance liquid chromatography-tandem mass spectrometry approach revealed TSPAN30, DDB1, TYMP, VDAC2, and DCXR as the top five candidate biomarkers for directly comparing samples of follicular adenoma and normal thyroid tissue [106].

The Tspan protein family can also be used as a biomarker of cancer-targeted therapy. The induction of autophagy gene ATG7 and the conversion of autophagy markers (LC3-I and LC3-II) and Tspan28, Tspan29, and Tspan30 in rotenone-treated prostate and breast cancer stem cells were detected by Western blotting. The results showed that rotenone could induce tumor stem cells to express Tspan28, Tspan29, Tspan30, and TSG101 [107, 108]. However, due to the limitation of experimental data and the presence of Tspan molecules on the surface of normal cells and tumor-related tissues, there are still some restrictions on the Tspan protein family as a biomarker.

## The role of the Tspan protein family in cancer treatment

The significant role of the Tspan protein family in tumor development and therapy cannot be overlooked. Tspan proteins located in the biofilm of tumor cells can serve as target molecules for drugs and antibodies. Additionally, Tspan proteins secreted by exosomes, which detach from the cell membrane, play crucial roles in recognition and signaling. This suggests the potential for drug encapsulation in exosomes targeted specifically to tumor cells, enhancing therapy efficiency and precision. Moreover, microRNAs can directly target Tspan genes, altering their expression levels. The diverse range of effectors within the Tspan protein family offers numerous possibilities for clinical treatment (Table 3). Currently, specific antibodies or RNA interference strategies are being utilized to modulate Tspan protein family-associated signaling, with some agents demonstrating significant results in animal studies.

**Table 3:** The role of the Tspan protein family in cancer treatment.

Member	Cancer type	Effect	Reference
Tspan1	Gastric cancer	miR-573 downregulated the expression of Tspan1	[41]
	Pancreatic cancer	Targeting molecules of miR454 and miR-573	[10, 124]
	Cervical cancer	MiR-361-3p downregulated the expression of Tspan1	[125]
Tspan3	Acute myeloid leukemia	Decrease the sensitivity of cancer cells to adriamycin	[118]
	Acute myeloid leukemia	MiR-570-3p downregulated the expression of Tspan3	[126]
Tspan8	Gastric cancer	Decrease the sensitivity of cancer cells to cisplatin, 5-FU, and adriamycin	[117]
	Gastric cancer	MiR-324-5P downregulated the expression of Tspan8	[137]
Tspan9	Gastric cancer	Decrease the sensitivity of cancer cells to 5-FU	[117]
	Hepatocellular carcinoma	Hsa-miR-9-5p downregulated the expression of Tspan9	[72]
Tspan12	NSCLC	MiR-196b-5p downregulated the expression of Tspan12	[128]
Tspan13	Papillary thyroid carcinoma	Targeting molecules of miR-369-3p	[21]
	Breast cancer	miR-4732-5p downregulated the expression of Tspan13	[129]
Tspan17	Glioblastoma multiforme	MiR-378a-3p downregulated the expression of Tspan13	[129]
Tspan24	Hepatocellular carcinoma	Correlate with sorafenib resistance	[12]
	Breast cancer	MiR-124 downregulated the expression of Tspan24	[130]
Tspan27	Gastric cancer	MiR-197 downregulated the expression of Tspan27	[131]
Tspan28	Esophageal cancer	APW can bind to Tspan28 and inhibit its function	[110]
	Acute myeloid leukemia	Increasing cellular resistance to conventional chemotherapy	[118]
Tspan29	Acute myeloid leukemia	Increasing cellular resistance to Ara-chemotherapy	[118]
	B-cell lymphomas	Associated with cellular chemotherapy resistance	[119]
	Gastric cancer	Targeting molecules of miR-155-5p	[132]
	Gastric cancer	MiR-142-5p downregulated the expression of Tspan29	[133]
	Prostate cancers	MiR-518f-5p downregulated the expression of Tspan29	[134]
	Breast cancers	MiR-518f-5p downregulated the expression of Tspan29	[134]

### The role of the Tspan protein family in drug therapy

The Tspan protein family plays a crucial role in the pharmacological treatment of tumors, serving as both targets for antitumor drugs and antibodies and as therapeutic targets to overcome drug resistance and enhance drug sensitivity. Clinically approved drugs often have limitations in target accumulation due to their lack of specificity, resulting in modest efficacy and numerous adverse effects. To enhance their potential, researchers utilize various targeting molecules, with Tspan proteins exhibiting significant promise in this regard [109]. Yue et al. were the first to demonstrate the binding of Andrographis paniculata water (APW), including its components Andrographis paniculata Lactone, Andrographis paniculata A, and Andrographis paniculata C, to Tspan28, resulting in the inhibition of its function. These findings suggest a potential relationship between these compounds and the anti-metastatic activity of APW in esophageal cancer [110].

The Tspan protein family is exposed on the surface of the cell membrane, which permits the immobilization of antibodies that can act as targeting molecules for related monoclonal antibodies. There is substantial evidence supporting the inhibition of tumor growth and potential induction of partial or complete remission through the targeting of Tspan proteins with antibodies. Overexpression of Tspan8 in rats promotes angiogenic activity and supports tumor growth, and an anti-rat Tspan8 monoclonal antibody effectively inhibits this process [111]. Four fully human antibodies with distinct complementarity-determining regions (CDRs) were isolated from a synthetic human antibody library using phage display technology. Among these antibodies, the one exhibiting the highest affinity for binding to Tspan8-LEL was selected. The antibody was found to specifically recognize amino acid residues 140–205 of Tspan8-LEL in a conformation-dependent manner, and these findings demonstrated that Tspan8-LEL may be a potential therapeutic target for cellular invasion of metastatic colorectal cancer cells [112]. The anti-Tspan24 antibodies inhibited metastatic spreading and primary tumor growth in a mouse model of human tumors, and they possessed the ability to destroy the ability to disrupt the complex between Tspan24 and α3β1 integrin [111]. Tspan26 has been suggested as a potential target for the treatment of B-cell lymphomas using radiolabeled anti-Tspan26 antibodies [111]. By introducing the E430G mutation into the humanized Tspan26 monoclonal antibody, IgG1, and utilizing intermolecular Fc-Fc interactions upon binding to cell-surface antigens, more efficient formation of IgG hexamers is achieved. These modified antibodies exhibit stronger complement-dependent cytotoxicity (CDC) activity compared to monoclonal antibodies lacking the E430G mutation, making them potential candidates for the treatment of B-cell malignancies [113]. Tspan28 is also an anticancer target, and Tspan28 signaling is involved in the development of solid tumors development and is associated with the aggressiveness of B-cell lymphomas, Tspan28 has been investigated as an antiviral and/or anticancer agent, and a number of anti-Tspan28 monoclonal antibodies have been developed, notably mAb5A6, which is anti-invasive and metastatic in triple-negative breast cancer cells [114]. Tspan28-EC2 can also be used as a natural product and as synthetic compounds as targets [114]. Intravenous injection of anti-Tspan29 monoclonal antibody ALB6 positively affected subcutaneous transplanted tumors in nude mice with human gastric carcinoma, with a reduction in tumor volume of 60–70% in the treated group of mice as compared to the control group, while at the same time, a significant reduction in cell proliferation and angiogenesis and an increase in apoptotic signals were observed [111].

Tspan proteins are capable of increasing drug resistance and can be inhibited to overcome resistance or increase drug sensitivity. Several studies have identified Tspan1 as a significant contributor to the acquired resistance of tumor cells to conventional chemotherapy [115]. miR-155 exerts its influence by targeting Tspan5, leading to increased stemness and resistance to Decitabine (DCA) in breast cancer cells [116]. Tspan8 is implicated as a drug-resistant protein in gastric cancer cells. However, silencing Tspan8 expression enhances the sensitivity of cancer cells to cisplatin, 5-fluorouracil (5-FU), and adriamycin. Tspan8 activates the Wnt/β-conjugated protein pathway by binding to NOTCH2, which increases the expression and accumulation of β-conjugated proteins in the nucleus and creates multidrug resistance. Tspan8 inhibitors may be developed as an adjuvant therapy for gastric cancer to reduce the cancer cells’ resistance [117]. Qi et al. discovered that 5-fluorouracil (5-FU)-resistant gastric cancer cells exhibit high expression of Tspan9 and P55, which binds to PIK3R3 and subsequently inhibits the activation of the PI3K/AKT/mTOR pathway. This inhibition promotes autophagy, contributing to 5-FU resistance. Sorafenib, a vascular endothelial growth factor (VEGF) inhibitor, is a first-line agent for treating hepatocellular carcinoma, and its monotherapy has become the standard systemic therapy for advanced cases. Strong correlations have been observed between the levels of Tspan24 in hepatocellular carcinoma cells and resistance to sorafenib [118]. Tspan28-positive AML (Acute myeloid leukemia) cells are 30–50% more resistant to conventional chemotherapy, and Tspan29 has also been shown to regulate the development of leukemogenesis and resistance to chemotherapy. Tspan29-positive AML cells are more likely than Tspan29-negative AML cells are more resistant and migratory to Ara-C chemotherapy [118]. Tspan3 has been shown to be upregulated in both adriamycin-resistant AML samples and cell lines [118]. In the tumor microenvironment, Tspan29 is responsible for the crosstalk between BMMSCs (bone marrow mesenchymal stem cells) and breast cancer cells, which leads to chemoresistance [119]. Consistent with this, studies in small-cell lung cancer cells have shown that ectopic overexpression of Tspan29 enhances β1 integrin-mediated cell adhesion to extracellular matrix fibronectin, a fibronectin that is involved in cell adhesion-mediated resistance to drugs. Koch et al. found that when B-cell lymphomas isolate Dox (doxorubicin) isolated within Tspan29-positive exosomes and then exported it outside the cell, chemoresistance developed [119].

Several studies have demonstrated the potential of cancer treatment through the upregulation of Tspan proteins. Tspan27, in particular, has been identified as a metastasis suppressor that could be employed to halt breast cancer metastasis. However, this strategy is only applicable to breast cancer patients with low levels of Tspan27 [120]. The Tspan protein family, as a specific surface molecule present on exosomes, can selectively bind to specific cells, enabling exosomes to exhibit natural cell targeting abilities. Intravenous administration of exosomes loaded with paclitaxel demonstrates potent antitumor efficacy [121]. In recent years, exosomes have emerged as a highly effective drug delivery system characterized by low immunogenicity, high biocompatibility, and potent therapeutic capabilities. These vesicles enable the organic loading of nucleic acids, peptides, lipids, or small molecules with therapeutic functions, facilitating their targeted delivery to specific cell types or tissues in the body, particularly tumor tissues [122].

Numerous studies have acknowledged and elucidated the targeting properties of Tspan proteins. However, several challenges persist in achieving effective clinical treatment, and the research for clinical applications is still in its early stages. There is still a considerable journey ahead before biologics targeting Tspan proteins can be implemented in clinical practice.

#### Mechanism summary

Antibodies targeting specific Tspan proteins can inhibit their function and tumor growth. For example, anti-Tspan8 antibody has been shown to inhibit tumor growth in mouse models.

Certain natural products, such as Andrographis paniculata water (APW), can bind to Tspan proteins and suppress their activity. Overexpression of Tspan1 contributes to the acquired resistance of tumor cells to conventional chemotherapy. Silencing Tspan5 expression enhances the sensitivity of cancer cells to Decitabine (DCA) in breast cancer cells. Tspan8 promotes drug resistance in gastric cancer cells, but silencing its expression enhances their sensitivity to cisplatin, 5-fluorouracil (5-FU), and adriamycin. Tspan9 promotes 5-fluorouracil (5-FU) resistance in gastric cancer cells. Sorafenib resistance is associated with high levels of Tspan24 in hepatocellular carcinoma (HCC) cells. Tspan28-positive acute myeloid leukemia (AML) cells are more resistant to chemotherapy.Tspan29 is involved in the development of leukemogenesis and resistance to chemotherapy. Tspan proteins present on exosomes can selectively bind to specific cells, enabling exosomes to exhibit natural cell targeting abilities.

### The role of the Tspan protein family in microRNA

MicroRNAs (miRNAs) and the Tspan protein family form a tightly knit alliance, with miRNAs playing crucial roles in regulating key pathways associated with tumorigenesis [58]. Several miRNAs have been identified as direct targets of Tspan genes or mRNAs, often leading to the downregulation of Tspan gene expression and influencing tumor progression. This highlights the potential of miRNAs as therapeutic targets for cancer treatment [123].

MiR454 and miR-573 affected the proliferation, autophagy, migration, and invasion of pancreatic cancer cells by targeting Tspan1 [10, 124]. In gastric cancer, overexpression of miR-573 similarly downregulated the expression of Tspan1, which inhibited the proliferation and invasion of cancer cells [41]. miR-361-3p can target Tspan1 and negatively regulate Tspan1 expression, and the overexpression of miR-361-3p inhibited cervical cancer cell survival, migration, and invasion [125]. In contrast, miR-570-3p has been found to inhibit the progression of aggressive AML by downregulating Tspan3. Moreover, CIRC_0004136 acts as a miR-570-3p sponge, regulating the expression of Tspan3 [126]. Additionally, miR-155 has been demonstrated to induce stemness and DCA resistance of breast cancer cells by inhibiting the direct target gene Tspan5 [116]. It was found that miR-324-5P reduced gastric cancer cell survival and induced apoptosis in vitro by downregulating the expression of Tspan8 in human gastric cancer, in addition, miR-324-5p overexpression significantly inhibited gastric cancer cell tumorigenesis in vivo, as evidenced by a smaller tumor volume than that of the control group [127]. Hsa-miR-9-5p has been found to be significantly upregulated in patients with hepatocellular carcinoma and is associated with poor prognosis. Additionally, hsa-miR-9-5p-mediated downregulation of Tspan9 expression is correlated with a poor prognosis of hepatocellular carcinoma, immunologically related signaling pathways, and immune infiltration of the tumor [72]. Hypomethylation of the promoter region of the miR-196b-5p gene in NSCLC promoted the expression of miR-196b-5p, resulting in a significant upregulation of miR-196b-5p expression in NSCLC tissues, and the upregulated miR-196b-5p promoted the migration of lung cancer cells by directly targeting the tumor suppressor genes GATA6 and Tspan12, proliferation; cell cycle and the expression of both GATA6 and Tspan12 was downregulated in NSCLC tissues and negatively correlated with that of miR-196b-5p [128]. Tspan13 is a direct target of miR-369-3p, and overexpression of miR-369-3p significantly inhibited the proliferation and promoted apoptosis of PTC (papillary thyroid carcinoma) cells, and silencing of Tspan13 inhibited the effect of miR-369-3p on PTC cells [21]. MiR-4732-5p can also directly target the 3′-UTR of Tspan13 and inhibit the expression of Tspan13 at both the mRNA and protein levels, it was also found that miR-4732-5p may function as a tumor suppressor in the initial stages of breast cancer, while plays a tumor-promoting role by targeting Tspan13 during breast cancer progression [129]. MiR-378a-3p inhibits the progression of GBM by decreasing the expression of Tspan17, promotes apoptosis, and reduces proliferation, migration, and invasion, and thus may be a potential target for the treatment of GBM [58]. MiR-124 plays an important role in inhibiting invasion and metastasis of breast cancer cells by directly targeting the 3′-UTR of Tspan24 mRNAs and inhibiting its mRNA expression and protein translation [130]. In gastric cancer, miR-197 targets the 3′-UTR of Tspan27 and thus plays an inhibitory role against Tspan27 [131]. HSA-miR-217 and its target gene sirtuin 1 act as metastasis suppressor and promoter genes, respectively, in NSCLC, and they form an hsa-miR-217/sirtuin1/p53/Tspan27 metastasis regulatory pathway that plays an important role in brain metastasis of NSCLC [123]. In H. pylori infection-associated gastritis and gastric cancer, Tspan29, MST1R, and ADAM10 are the most likely targets of miR-155-5p [132]. LSD1 (histone lysine-specific demethylase 1) expression was significantly higher in metastatic gastric cancer tissues than in normal tissues, and it was also found that LSD1 elevated intracellular miR-142-5p, leading to downregulation of the expression of Tspan29, a novel target of miR-142-5p, which in turn promotes gastric cancer migration [133]. MiR-518f-5p decreased the expression of Tspan29 in prostate and breast cancers and led to increased cell migration [134].

MicroRNAs can also serve as targets of Tspan genes or mRNAs. For instance, CircTspan24 targets miR-30d-5p to upregulate the expression of GLI2(Glioma cancer-related gene homologous protein 2) and promote the malignant proliferation of lung adenocarcinoma and promote the malignant proliferation of lung adenocarcinoma [135]. Increasing evidence suggests that there is aberrant expression of miR-203 in tumor stem cells, and it has been found that Tspan27 upregulates the expression of miR-203, which in turn regulates the expression of FZD2 [136]. The relationship between microRNAs and the Tspan protein family as well as their mechanism of action still requires more in-depth study, whether microRNAs are potential targets for cancer therapy, and how to translate this knowledge into clinical applications remains an open question.

## Conclusions and future directions

Despite the growing number of studies revealing the importance of the Tspan protein family in tumor initiation and progression, the intricate nature of their biological functions necessitates further investigation into their specific molecular mechanisms and regulatory diversity. The Tspan protein family exerts a critical role in cancer by modulating the expression of oncogenes, influencing signaling pathways relevant to cancer development and progression, and regulating miRNA generation and signaling pathways. Moreover, preliminary data yield inconsistent findings regarding the role of Tspan proteins in either promoting or inhibiting tumorigenesis and progression. These discrepancies could potentially stem from variations in the composition of TEMs and the interacting protein chaperones.

Internalization of the Tspan protein family into intracellular vesicles and intercellular signaling via exosomal/extracellular vesicles represents a significant regulatory mechanism that can either impede or promote tumor metastasis. Tspan proteins, abundant in the membrane of exosomal/extracellular vesicles, not only engage in the signaling process by interacting with receptor proteins on the surface of recipient cells but also determine the sorting and selection of exosomal/extracellular vesicles upon detachment from the cell membrane. Both extracellular vesicles and intracellular multivesicular body vesicles encapsulate signaling molecules (proteins, microRNAs) during the sorting and selection process. With the increasing research on this family, the emergence of drug-targeted therapies against Tspan proteins has provided new opportunities for targeted cancer therapy. However, several challenges must be addressed. Firstly, as integral components of multiprotein complexes, Tspan proteins are influenced by various interacting elements and exhibit context-dependent functional roles. Secondly, the structural similarity among family members poses challenges in targeted therapy. These factors collectively impact the application of Tspan proteins as molecular markers and therapeutic targets. In the future, extensive and systematic investigations are warranted to elucidate the involvement of the Tspan protein family in cancer and its associated signaling pathways, establish interaction networks, uncover their precise mechanisms of action in cancer, and foster innovative approaches for targeted cancer therapy. Furthermore, efforts should be directed towards identifying natural bioactive compounds capable of replacing small molecule inhibitors to mitigate toxic side effects on normal cells. Simultaneously, microRNAs targeting the Tspan protein family offer a novel avenue for targeted cancer therapy, especially in conjunction with radiation therapy, chemotherapy, and other treatment modalities.

While current studies have only scratched the surface of Tspan’s involvement in tumorigenesis and progression, an increasing body of data is progressively unraveling its enigmatic nature. As our comprehension of Tspan’s functionality expands, it will pave the way for novel diagnostic and therapeutic approaches to precisely target interventions in the future.

## Data Availability

The authors confirm that the data supporting the findings of this study are available within the article.
